# Carbapenem-Resistant *Klebsiella pneumoniae* in Large Public Acute-Care Healthcare System, New York, New York, USA, 2016–2022

**DOI:** 10.3201/eid2910.230153

**Published:** 2023-10

**Authors:** Jennifer Lee, Subin Sunny, Elizabeth Nazarian, Mary Fornek, Marie Abdallah, Briana Episcopia, Marie-Claire Rowlinson, John Quale

**Affiliations:** New York City Health and Hospitals/Kings County, New York, New York, USA (J. Lee, S. Sunny, M. Abdallah, B. Episcopia, J. Quale);; New York City Health and Hospitals/Central Office, New York (M. Fornek);; Wadsworth Center, New York State Department of Health, Albany, New York, USA (E. Nazarian, M.-C. Rowlinson)

**Keywords:** Klebsiella pneumoniae, carbapenems, Beta-lactam resistance, bacteria, New York, United States, antimicrobial resistance, carbapenem-resistant Enterobacterales, CRE

## Abstract

Controlling the spread of carbapenem-resistant Enterobacterales is a global priority. Using National Healthcare Safety Network data, we characterized the changing epidemiology of carbapenem-resistant *Klebsiella pneumoniae* (CRKP) in a large public health system in New York, New York, USA. During 2016–2020, CRKP cases declined; however, during 2021–June 2022, a notable increase occurred. Of 509 cases, 262 (51%) were considered community-onset, including 149 in patients who were living at home. Of 182 isolates with proven or presumptive (ceftazidime/avibactam susceptible) enzymes, 143 were serine carbapenemases; most confirmed cases were *K. pneumoniae* carbapenemase. The remaining 39 cases were proven or presumptive metallo-β-lactamases; all confirmed cases were New Delhi metallo-β-lactamases. After 2020, a marked increase occurred in the percentage of isolates possessing metallo-β-lactamases. Most patients with metallo-β-lactamases originated from long-term care facilities. An aggressive and universal program involving surveillance and isolation will be needed to control the spread of CRKP in the city of New York.

Carbapenems have long served as tools in our arsenal against infections caused by multidrug resistant gram-negative bacteria; as such, the spread of carbapenem-resistant Enterobacterales (CRE) represents a serious public health threat. Because CRE infections are associated with poorer clinical outcomes and have limited treatment options, the Centers for Disease Control and Prevention has labeled CRE as an urgent threat (*1*,*2*).

Since its identification in 2001, *Klebsiella pneumoniae* carbapenemase (KPC) has been the predominant carbapenemase in CRE in medical centers across the United States (*3*,*4*). This carbapenemase has been also problematic in long-term care facilities throughout the United States (*5*,*6*). Although isolates with class B carbapenemases, particularly the New Delhi metallo-β-lactamase (NDM), are endemic in several regions of the world, they have been somewhat unusual in the United States, accounting for only ≈10% of identified carbapenemases (*4*,*7*). Given their greater resistance to β-lactam antibiotics (e.g., β-lactam/β-lactamase–inhibitor antibiotics, including ceftazidime/avibactam), the spread of isolates harboring metallo-β-lactamases is particularly worrisome (*8*).

For more than a decade, KPC has been the predominant carbapenemase found in the New York, New York, USA (New York City), region (*9*,*10*). However, in 2018, sporadic instances of residents of long-term care facilities in New York harboring NDM-possessing *K. pneumoniae* were reported (*10*,*11*). In this article, we document the changing epidemiology of carbapenem-resistant *K. pneumoniae* in hospitals across a large public health system in New York City.

## Methods

The New York City Health and Hospitals Enterprise consists of 11 acute care medical centers, of which 5 are level I trauma centers, 1 is a level II trauma center, and 1 is a level II pediatric trauma center. New York City Health and Hospitals serves >1.2 million patients annually. In 2022, >164,000 inpatient visits and 1 million emergency department visits occurred. All of the hospitals are public hospitals that serve patients primarily of low socioeconomic status; health equity barriers involving pregnancy, asthma, hypertension, diabetes, aging and frailty, substance use disorder, mental health, and violence are well-recognized in this population. Throughout the system, all patients with CRE are placed on strict contact precautions; completing a rigorous protocol is required to discontinue those precautions.

For this study, we obtained listings of patients with laboratory-identified carbapenem-resistant *K. pneumoniae* from the Centers for Disease Control and Prevention National Healthcare Safety Network database. Only 1 patient isolate per calendar year was included in the study. Following National Healthcare Safety Network definitions, we considered cases community-onset if the isolate was recovered in the outpatient setting or during the first 3 days of admission and healthcare facility–onset if recovered after 3 days of admission. We considered cases to be community-onset healthcare facility–associated if the patient had been discharged from an inpatient facility within 90 days (*12*,*13*) or if the patient had dialysis-dependent end-stage renal disease. We retrospectively reviewed medical records, including taking note of whether the patient was admitted from home, from a long-term care facility, or from another acute-care hospital. In addition, we documented the results of carbapenemase testing and ceftazidime/avibactam susceptibility testing performed by the clinical laboratory, if available. We also included results of carbapenemase testing of isolates submitted to the New York State Department of Health. We considered isolates in which KPC or OXA-48 were detected as proven serine carbapenemase producers and isolates susceptible to ceftazidime/avibactam to be presumptive serine carbapenemase producers (*14*). Similarly, we considered isolates with NDM detected proven metallo-β-lactamase producers and isolates that were resistant to ceftazidime/avibactam presumptive metallo-β-lactamase producers (*14*).

We used the Student t-test to compare continuous values and χ^2^ analysis to compare categorical values. This study was approved by the SUNY Downstate Medical Center Institutional Review Board and the Health and Hospitals System to Track and Approve Research program.

## Results

During January 1, 2016–June 30, 2022, a total of 509 patients with carbapenem-resistant *K. pneumoniae* were identified. Of the 509 patients, 306 (60.1%) were men and 203 (39.9%) women; 193 (38%) were Black, 114 (22%) were Hispanic, 106 (21%) were White, and 50 (10%) were Asian. Major sources of positive cultures were genitourinary tract (52%), respiratory tract (21%), bloodstream (12%), and skin or soft tissue (11%). Of the 509 patients, 262 were considered to have community-onset positive cultures; sources were genitourinary tract (65%), respiratory tract (11%, of which 38% were transtracheal aspirates), bloodstream (11%), and skin or soft tissue (7%). The remaining 247 patients were considered to have hospital-onset cultures; sources were genitourinary tract (38%), respiratory tract (30%, of which 40% were transtracheal aspirates), bloodstream (13%), and skin or soft tissue (38%). Of note, only 4 of the 29 transtracheal isolates in the hospital-onset group occurred after March 2020, suggesting that the COVID-19 pandemic did not influence the results.

Of the 262 patients with community-onset cultures, 149 lived at home and 108 in a long-term care facility; 5 were transfers from another acute-care hospital. Of the 149 patients that resided at home, 68 were considered to have community-onset healthcare facility–associated positive cultures (61 patients had been discharged as an inpatient within 90 days and 7 were on maintenance hemodialysis). Of the 247 patients with hospital-onset cultures, 133 lived at home, 103 lived in a long-term care facility, and 11 were transfers from another acute-care facility.

**Figure 1:**
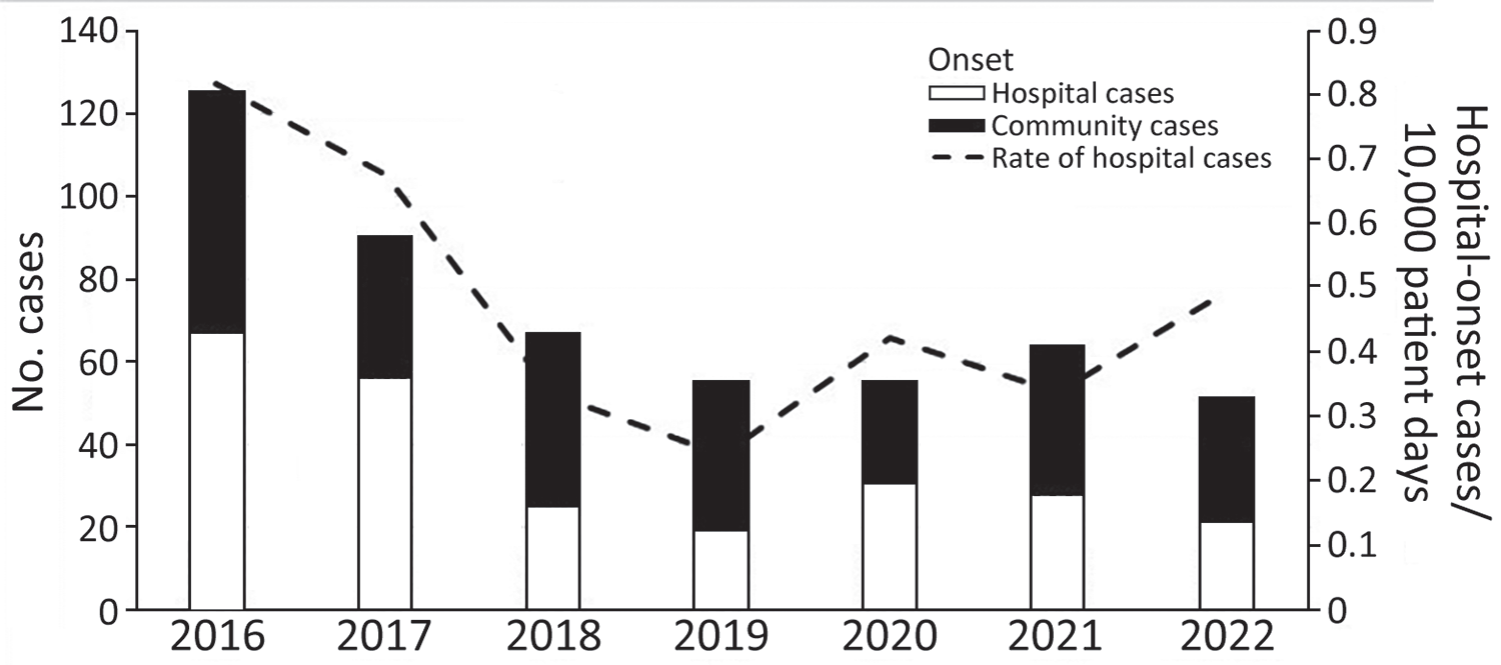
Number of community-onset cases versus hospital-onset cases and rate of hospital-onset infections with carbapenem-resistant *Klebsiella pneumoniae*, New York, New York, USA, 2016–2022.

Cases declined steadily during 2016–2020, but this trend was reversed during the latter part of 2021 and early 2022, when a noticeable increase in the number of cases occurred (Figure 1). The rates of hospital-onset cases followed a similar trend (Figure 1). Correlated with this increase was a marked change in patients’ place of residence before the culture. During 2016–2020, a total of 143 (36.3%) of 394 patients with carbapenem-resistant *K. pneumoniae* originated from long-term care facilities. During the next 18 months, however, 68 (59.1%) of 115 patients originated from long-term care facilities (p<0.0001).

**Figure 2:**
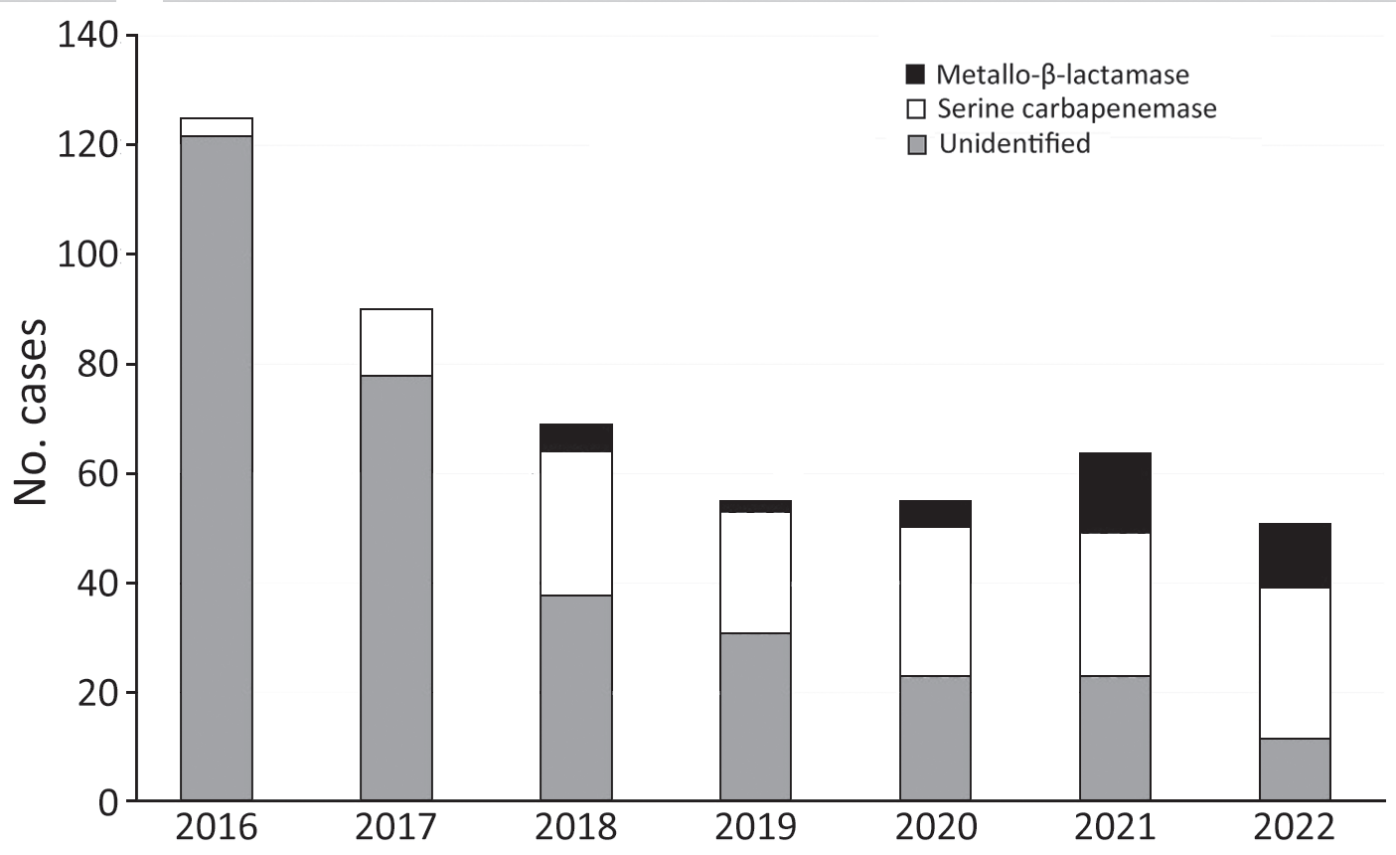
Number of proven and presumptive serine and metallo-β-lactamases identified from carbapenem-resistant *Klebsiella pneumoniae*, New York, New York, USA, January 2016–June 2022.

Carbapenemase identification or ceftazidime/avibactam susceptibility testing was performed in 182 cases. A total of 143 patients had serine carbapenemases (proven KPC in 43 and OXA-48 in 3 patients; 97 patients with susceptibility to ceftazidime/avibactam). There were 39 patients with metallo-β-lactamases (proven NDM in 15 and resistance to ceftazidime/avibactam in 24). Concomitant with the trends we have noted, a marked shift in the type of carbapenemases also occurred during the study period (Figure 2). During 2016–2020, only 12 (11.8%) of 102 patients were found to have metallo-β-lactamases. During January 2021–June 2022, however, 27 (33.8%) of 80 patients had metallo-β-lactamases (p = 0.0007 compared with 2016–2020). In all, 67 patients with serine carbapenemases originated from long-term care facilities; they resided in 30 different facilities before the culture. Twenty of the patients with metallo-β-lactamases originated from long-term care facilities, and they resided in 13 different facilities.

## Discussion

This retrospective review of patients with carbapenem-resistant *K. pneumoniae* revealed several notable observations. First, it is disconcerting that approximately half of the patients had community-onset cultures; 31% of patients with community-onset isolates lived at home, were not on hemodialysis, and had not been recently hospitalized. Previous studies have indicated cases of community-onset CRE are rather unusual, accounting for ≈10% of cases (*15*,*16*).

The reason for the high percentage of community-onset CRE seen in our study might be multifactorial. The New York City Health and Hospitals acute care hospitals serve predominantly urban communities of low socioeconomic status. High rates of community-associated methicillin-resistant *Staphylococcus aureus* and *Clostridioides difficile* infections have been reported in communities of low socioeconomic status and have been attributed to poverty and overcrowding (*17*,*18*). Increasing rates of community-onset isolates possessing extended-spectrum β-lactamases have been well documented (*19*). Healthcare exposure might be a key factor, but studies in Europe have also documented overcrowding, low socioeconomic status, and environmental and agricultural factors in the spread of extended-spectrum β-lactamase–possessing isolates in the community (*20*,*21*). Similar factors might promote the spread of carbapenemase-producing pathogens in the community. Previous studies have documented contamination of the areas surrounding hospitals, including in New York City, with carbapenemase-producing pathogens (*22*,*23*). Colonization of animals, including companion animals, might also be a potential source for spread of carbapenemase-producing bacteria in the community (*24*–*26*). Further study is needed to assess the contribution of healthcare-related factors, socioeconomic factors, and environmental factors in the community spread of carbapenem-resistant *K. pneumoniae* in the New York City region.

Second, we observed an increasing number of patients with carbapenem-resistant *K. pneumoniae* after the onset of the COVID-19 pandemic. This increase parallels the increase in methicillin-resistant *S. aureus*, *C. difficile*, and other hospital-acquired infections seen during the pandemic (*27*–*29*). Again, this finding is likely multifactorial. Increased antibiotic use, particularly of cephalosporins, early in the pandemic has been documented and was likely a contributing factor (*28*,*30*). Isolation of CRE has been associated with prolonged hospitalization; during the pandemic, carbapenem resistance among Enterobacterales was recovered in 4.2% of patients who had been hospitalized for 15–28 days but increased to 19% for stays beyond 28 days (*31*). During the pandemic, the emphasis on respiratory precautions might have overshadowed the need for contact precautions; issues regarding hand hygiene compliance and shortages of personal protective equipment might have been contributing factors.

Third, we observed a marked increase in metallo-β-lactamases since 2021 in this region. Before 2018, it was distinctly unusual for a carbapenemase other than KPC to be recovered in New York City (*10*). However, in 2018, reports of a small number of patients possessing the metallo-β-lactamases NDM, all residing in long-term care facilities in the region, emerged (*10*,*11*). Since then, carbapenem-resistant *K. pneumoniae* with NDM (or resistant to ceftazidime-avibactam) have spread, now accounting for one third of all isolates. Most patients in our study with presumptive or proven NDM-possessing isolates originated from long-term care facilities, indicating establishment in these centers as well. Our data provide an accurate glimpse of the status of carbapenemase producers in long-term care facilities in New York City. Of the existing 158 long-term facilities in the Bronx, Brooklyn, Manhattan, and Queens, we included patients from 67 sites in this study. Finally, reliance on the new β-lactam/β-lactamase inhibitors to treat CRE in our region can no longer be assumed, and carbapenemase identification or susceptibility testing is now essential.

The first limit of this retrospective study is that the number of cases of community-onset CRE might be underestimated, because recognition of the isolate in cases classified as hospital-onset simply might have been delayed. Also, detailed characterization of the patients with community-onset isolates is needed to determine the extent of healthcare exposure before the identification of CRE. We did not conduct statistical evaluation of the changing rates. Finally, the carbapenemase was identified in only a small percentage of isolates. We relied on ceftazidime/avibactam susceptibility to distinguish between serine and metallo-enzymes. Although unusual, mutations in the carbapenemase KPC might lead to resistance to ceftazidime/avibactam; however, these mutations often restore carbapenem susceptibility (*32*).

In conclusion, the number of community-onset cases, the increasing overall numbers, and the emergence of NDM-possessing carbapenem-resistant *K. pneumoniae* identified in this study are concerning. Aggressive and universal surveillance and isolation measures involving both acute-care and long-term care facilities, such as those implemented in Israel (*33*), will likely be needed to control further spread of these pathogens.
